# Carbon Monoxide Generation, Heme Turnover, and Lipid Peroxidation During Heme-Mediated LDL Oxidation: Association of HO-1, Hemopexin, and A1M with LDL

**DOI:** 10.3390/biom16101500

**Published:** 2026-10-15

**Authors:** Dávid Pethő, Gergő Kalló, Éva Csősz, István Bak, Magnus Gram, József Balla, György Balla, Tamás Gáll

**Affiliations:** 1 Department of Internal Medicine, Division of Nephrology, Faculty of Medicine, University of Debrecen, H-4032 Debrecen, Hungary; 2 HUN-REN–UD Vascular Biology and Myocardium Pathophysiology Research Group, Hungarian Academy of Sciences, University of Debrecen, H-4032 Debrecen, Hungary; 3 Department of Biochemistry and Molecular Biology, Faculty of Medicine, University of Debrecen, H-4032 Debrecen, Hungary; 4 Department of Pharmacodynamics, Faculty of Pharmacy, University of Debrecen, H-4032 Debrecen, Hungary; 5 Department of Clinical Sciences Lund, Pediatrics, Lund University, 221 84 Lund, Sweden; 6 Department of Pediatrics, Faculty of Medicine, University of Debrecen, H-4032 Debrecen, Hungary

**Keywords:** low-density lipoprotein, heme oxygenase-1, LDL oxidation, carbon monoxide, hemopexin, alpha-1-microglobulin, mass spectrometry

## Abstract

Heme promotes oxidative modification of low-density lipoprotein (LDL), a process implicated in atherosclerosis. However, the fate of heme during LDL oxidation and the mechanisms governing its degradation remain poorly understood. We investigated heme catabolism during heme/H_2_O_2_-induced LDL oxidation and identified LDL-associated proteins potentially involved in this process. Lipid peroxidation, heme Soret absorbance, and carbon monoxide (CO) production were assessed. Heme induced lipid peroxidation, loss of Soret signal, and CO generation, whereas CuSO_4_-mediated oxidation induced lipid peroxidation without detectable CO, supporting the association of CO production with heme-dependent oxidation. Targeted and data-independent proteomics and immunological analyses identified heme oxygenase-1 (HO-1), hemopexin (HPX), and alpha-1-microglobulin (A1M) in LDL. HO-1 inhibition reduced CO production and delayed early heme loss and lipid peroxidation, although these effects diminished over time. DTPA similarly delayed early heme loss and lipid peroxidation and reduced CO production, whereas BHT strongly suppressed lipid peroxidation, preserved the heme Soret signal, and reduced CO production throughout oxidation. HPX attenuated heme-induced LDL oxidation and CO generation. These findings indicate that HO-1-associated iron release contributes to early phase heme-mediated LDL oxidation along with CO generation, and heme-associated iron without liberation also contributes the oxidative modification of LDL.

## 1. Introduction

Cardiovascular disease remains the leading cause of mortality worldwide, with atherosclerosis representing its principal underlying pathology [1,2,3]. The initiation and progression of atherosclerosis are closely linked to oxidative stress and the accumulation of oxidatively modified lipoproteins within the arterial wall. Among these, low-density lipoprotein (LDL) plays a central role, as oxidative modification of LDL enhances its uptake by macrophages, promotes foam cell formation, and triggers inflammatory responses that contribute to plaque development and progression [4,5,6].

Free heme is a potent pro-oxidant that is released during intravascular hemolysis, tissue injury, and the degradation of hemoproteins. Owing to its redox-active iron, heme can catalyze oxidative reactions that promote lipid peroxidation and oxidative modification of biomolecules [7,8]. Heme-induced oxidation of LDL has been implicated in vascular dysfunction and the progression of atherosclerosis [7,8]. Although heme-mediated lipid peroxidation is well documented, the relative contributions of reactive oxygen species, lipid-derived radicals, and endogenous antioxidant systems to LDL oxidation remain incompletely understood.

Heme oxygenase-1 (HO-1) is the inducible, rate-limiting enzyme responsible for heme degradation, generating biliverdin, carbon monoxide (CO), and ferrous iron [9]. Induction of HO-1 is generally considered a cytoprotective response to oxidative stress because it limits the accumulation of free heme while producing metabolites with antioxidant, anti-inflammatory, and cytoprotective properties [10,11]. Consequently, HO-1 has been widely regarded as an important defense mechanism against heme-induced oxidative injury [12,13]. However, whether HO-1 directly limits heme-induced LDL oxidation or whether LDL-associated proteins contribute to extracellular heme metabolism remains unclear.

CO is an endogenous gaseous signaling molecule with diverse biological functions [14] including inhibition of heme release from heme proteins [15], and a range of other protective effects [16,17]. Endogenous CO is produced predominantly through the enzymatic degradation of heme by HOs [9], while a smaller fraction is generated through non-enzymatic processes, including lipid peroxidation [18].

LDL particles contain numerous proteins beyond apolipoprotein B-100 (APOB-100) [19]. These proteins are thought to influence lipid metabolism, inflammation, oxidative processes, and lipoprotein function. Nevertheless, the association of proteins directly involved in heme metabolism with LDL has received little attention, and their potential contribution to the regulation of heme-induced oxidative modification of LDL remains unknown.

In the present study, we investigated the role of HO-1 in heme-induced lipid peroxidation of LDL and examined the protein composition of LDL with a particular focus on proteins involved in heme metabolism. We show that inhibition of HO-1 significantly attenuates, but does not completely prevent, heme-induced lipid peroxidation, consistent with a contributory but not exclusive role for HO-1 in this process. Furthermore, we provide the first evidence that HO-1 and heme scavenger protein alpha-1-microglobulin (A1M) are associated with LDL, expanding the current understanding of LDL composition and suggesting previously unrecognized mechanisms through which LDL may participate in heme metabolism and oxidative processes.

## 2. Materials and Methods

### 2.1. Reagents

Unless otherwise stated, we obtained all reagents from Sigma-Aldrich (St. Louis, MO, USA). HO-1-IN, Butylated hydroxytoluene (BHT), Diethylenetriaminepentaacetic acid (DTPA) were obtained from MedChemExpress (Bergkällavägen, Sollentuna, Sweden) and dissolved in Dimethylsulfoxide (DMSO).

### 2.2. Preparation of Low-Density Lipoproteins

Native LDL (nLDL) was obtained from EDTA-anticoagulated venous blood collected from healthy volunteers and isolated from plasma using gradient ultracentrifugation (Beckman Coulter, Brea, CA, USA). The plasma was adjusted to a density of 1.3 g/mL using potassium bromide. A two-layer density gradient was subsequently established in a Quick-Seal ultracentrifuge tube by overlaying saline onto 10 mL of plasma. Ultracentrifugation was conducted at 302,000× *g* for 2 h at 4 °C using a VTi 50.2 rotor. Following isolation, nLDL preparations were dialyzed against 0.9% NaCl solution and stored at 4 °C, and protein concentrations were measured using the Pierce BCA Protein Assay Kit (Thermo Scientific, Waltham, MA, USA).

### 2.3. Measurement of Lipid Peroxidation Products in LDL

For short-term oxidative modification of nLDL by heme and H_2_O_2_, nLDL (200 μg) in 0.9% NaCl was co-incubated with heme (10 μM) and H_2_O_2_ (75 μM). For experiments assessing the effects of HO-1 inhibition, hemopexin (HPX), A1M, iron chelation, or radical scavenging, nLDL (200 μg) was pre-incubated for 60 min with HO-1-IN (20 μM), an HO-1 inhibitor; hemopexin (20 μM); recombinant A1M (rA1M, 20 μM); DTPA (80 μM), an iron chelator; or BHT (10 μM), a radical scavenger, before oxidation was initiated by adding heme and H_2_O_2_. Oxidative modification of nLDL by CuSO_4_ was initiated by adding CuSO_4_ to a final concentration of 40 μM.

For long-term oxidative modifications, nLDL (200 μg) in 0.9% NaCl was incubated with heme (10 μM) or CuSO_4_ (40 μM) for 7 days.

For analysis of conjugated diene formation during short-term oxidative modification, samples were incubated in 96-well plates at 37 °C in a spectrophotometer following initiation of oxidation with heme (10 μM) and H_2_O_2_ (75 μM) or CuSO_4_ (40 μM). Absorbance was continuously monitored at 234 nm for 180 min. For experiments assessing the inhibitory effects of A1M and HPX, conjugated diene formation was determined after 45 min.

To assess thiobarbituric acid-reactive substances (TBARs) during short-term oxidative modification, nLDL (200 μg) was incubated with heme (10 μM) and H_2_O_2_ (75 μM) or CuSO_4_ (40 μM) for 45, 90, or 180 min. For experiments assessing the inhibitory effects of A1M and HPX, TBARs formation was determined after 45 min. Following the incubation for the indicated periods, 100 μL of thiobarbituric acid (TBA) reagent, containing 0.375 g 2-thiobarbituric acid, 2.08 mL HCl, and 15 mL of 10% trichloroacetic acid (TCA), brought to a final volume of 100 mL, was added to 50 μL of the nLDL oxidation reaction mixture. Samples were heated at 90 °C for 20 min, as previously described [20]. After cooling, the reaction products were extracted with 200 μL n-butanol, and the absorbance of the upper phase was measured spectrophotometrically at 532 nm. TBARs levels were calculated using a molar extinction coefficient of 1.56 × 10^5^ M^−1^ cm^−1^.

For long-term oxidative modification, nLDL (200 μg) was incubated with heme (10 μM) or CuSO_4_ (40 μM) for 7 days. Conjugated diene formation and TBARS levels were determined after 7 days of incubation as described above.

### 2.4. Quantification of Heme by Soret Band Absorbance at 405 nm

To assess heme degradation, heme (10 μM) and H_2_O_2_ (75 μM) were added to nLDL (200 μg), and heme content was monitored by measuring the Soret absorbance at 405 nm using UV–visible spectroscopy. Absorbance measurements were performed continuously for 180 min following initiation of the oxidation reaction with heme and H_2_O_2_.

### 2.5. Sample Preparation for Mass Spectrometry

Gel bands were excised and reduced with 20 mM dithiothreitol (Bio-Rad, Hercules, CA, USA) for 1 h at 56 °C. Subsequently, alkylation was carried out using 55 mM iodoacetamide (Bio-Rad, Hercules, CA, USA) for 45 min at room temperature in the dark. Proteolytic digestion was then performed overnight at 37 °C with stabilized, MS-grade, TPCK-treated bovine trypsin (AB Sciex, Framingham, MA, USA). Following digestion, the resulting peptides were extracted and concentrated to dryness using a SpeedVac concentrator (Thermo Scientific, Waltham, MA, USA). The dried peptides were subsequently reconstituted in 10 μL of 1% formic acid (VWR Ltd., Radnor, PA, USA) and prepared for LC-MS/MS analysis.

### 2.6. PRM-Based Targeted Proteomics

The amino acid sequence of human HO-1 (UniProt accession P09601) was evaluated to identify proteotypic peptides. Two peptides, TEPELLVAHAYTR and VQDSAPVETPR, were subsequently selected as targets for parallel reaction monitoring (PRM).

LC–MS/MS measurements were performed using an Easy-nLC 1200 nanoUPLC system (Thermo Fisher Scientific, Waltham, MA, USA) interfaced with an Orbitrap Fusion mass spectrometer (Thermo Fisher Scientific, Waltham, MA, USA). Prior to chromatographic separation, the samples were desalted using an ACQUITY UPLC M-Class Symmetry C18 trap column (20 mm × 180 μm, 5 μm particle size, 100 Å pore diameter; Waters, Milford, MA, USA). Peptide separation was subsequently achieved on an ACQ M-Class BEH130 C18 analytical column (150 mm × 75 μm, 1.7 μm particle size, 130 Å pore diameter; Waters, Milford, MA, USA) using a 30-min water–acetonitrile gradient at a flow rate of 300 nL/min.

The mobile phases consisted of LC-grade water containing 0.1% formic acid (buffer A) and LC-grade acetonitrile supplemented with 0.1% formic acid (buffer B), with both solvents obtained from VWR Ltd. (Radnor, PA, USA). The proportion of buffer B was initially increased from 10% to 30% over 19 min. This was followed by a rapid increase to 85% B over 1 min, which was maintained for an additional 4 min. The system was then returned to 5% buffer B over 1 min and equilibrated at this composition for 5 min.

For mass spectrometric detection, a Nanospray Flex nanoelectrospray ionization source (Thermo Fisher Scientific, Waltham, MA, USA) was operated in positive-ion mode at a spray voltage of 2.3 kV, with the ion-transfer tube maintained at 275 °C. PRM measurements were conducted using a targeted MS2 acquisition strategy. The precursor ions corresponding to TEPELLVAHAYTR and VQDSAPVETPR were monitored at *m*/*z* 599.81 and 500.59, respectively. Following precursor-ion isolation in the quadrupole, peptide fragmentation was performed using higher-energy collisional dissociation (HCD) at 35% normalized collision energy. The resulting fragment ions were acquired over an *m*/*z* range of 100–1600 using the Orbitrap analyzer at a resolution of 60,000, with an automatic gain control (AGC) target of 1.0 × 10^6^. Data acquisition was performed in positive-polarity mode.

PRM spectra were analyzed using the Skyline software (v25.1.0.237) [21].

### 2.7. DIA-Based Proteomics Analysis

Data-independent acquisition (DIA) proteomic measurements were performed using the same LC–MS instrumentation described above for the PRM experiments. Chromatographic separation was conducted at a flow rate of 300 nL/min. The gradient began with an increase in solvent B from 0% to 1% over 210 min, followed by a stepwise increase to 5% B over 3 min and subsequently to 25% B over 56 min. Solvent B was then raised to 38% over 11 min and further increased to 90% over 4 min, where it was maintained for 3 min. The column was subsequently re-equilibrated by reducing solvent B to 2% over 1 min and maintaining this composition for 12 min. MS1 spectra were acquired over an *m*/*z* range of 350–1200 at a resolution of 120,000. The precursor population was subsequently analyzed using DIA with 55 fixed isolation windows, each spanning 12 *m*/*z*. Fragmentation was performed by higher-energy collisional dissociation (HCD) using a normalized collision energy of 30%, generating the corresponding MS2 spectra. Fragment ions were measured with the Orbitrap analyzer over an *m*/*z* range of 145–1450 at a resolution of 30,000. The automatic gain control (AGC) target was set to 2.0 × 10^5^, with a maximum injection time of 60 ms.

Raw files were processed using DIA-NN (v2.2) [22] in library-free search mode. A human protein sequence FASTA database (20,420 reviewed entries) was downloaded from UniProtKB on 26 July 2025, and ‘FASTA digest’ and ‘Deep-learning spectra’ were enabled for in silico library generation. Other parameters included: Trypsin/P with a maximum of 2 missed cleavages; 3 maximum number of modifications; C-Carbamidomethylation, M oxidation and N-terminal acetylation were set as variable modifications. The XICs output was enabled and all other parameters were kept as default.

The DIA-NN PARQUET output files were imported into the DEF workflow [23]. Proteins identified with at least two sequences were accepted as hits.

### 2.8. Quantification of CO by Gas Chromatography

Carbon monoxide (CO) in the vial headspace was quantified using a GC-2010 gas chromatograph (Shimadzu, Kyoto, Japan). For each sample, 3 mL of headspace gas was withdrawn with a gastight syringe (SGE-10MDR-HSV) and introduced into the gas chromatograph through an MGS-5 gas sampler (Shimadzu, Kyoto, Japan) fitted with a calibrated 0.5 mL sample loop. Hydrogen was used as the carrier gas at a linear velocity of 30 cm/s. The injector was maintained at 55 °C and operated in split mode with a split ratio of 10:1. Gas separation was performed under isothermal conditions using a Varian Molsieve 5A PLOT column (30 m × 0.32 mm × 10 μm) (Agilent Technologies, Inc., Santa Clara, CA, USA) maintained at 65 °C. Detection was achieved with a thermal conductivity detector (TCD) operated at a current of 90 mA and maintained at 70 °C. Headspace CO concentrations were determined at two experimental time points, 60 min and 7 days after sample preparation.

### 2.9. Western Blot

To analyze the presence of HO-1 and A1M in nLDL preparations, 40 μg of nLDL were run on 12% Tris-glycine SDS-PAGE gels. After blocking, membranes were probed for antibodies against rabbit A1M (Abcam, Cambridge, UK; dilution 1:2500) and rabbit HO-1 (Proteintech Group, Manchester M3 3WF, UK; dilution 1:8000) at 4 °C overnight, followed by incubation with horse-radish peroxidase (HRP)-conjugated goat anti-rabbit antibody (Cell Signaling Technologies, Danvers, MA, USA) at room temperature for 1 h. The antigen–antibody complex was detected by a WesternBright Quantum HRP substrate (Advansta, Menlo Park, CA, USA) and detected with Ibright F1500 Imaging System (Thermo Fisher Scientific, Waltham, MA, USA).

### 2.10. A1M ELISA

Human LDL samples from healthy donors were collected and analyzed using Human alpha 1 microglobulin ELISA kit (ab108884, Abcam, Cambridge, UK). Samples were diluted (dilution 1: 10,000 in PBS) and the measurement were carried out according to the manufacturer’s guide. A1M expressions were normalized to protein content determined using BCA (Pierce BCA Protein Assay Kit, Thermo Fisher Scientific, Waltham, MA, USA).

### 2.11. Immunofluorescent Staining

LDL samples isolated from healthy donors were transferred onto poly-L-lysine-coated coverslips and incubated for 2 h at 37 °C. For HO-1 immunostaining, the samples were fixed with 4% paraformaldehyde for 15 min, then permeabilized with 0.1% Triton X-100 in phosphate-buffered saline (PBS) for 10 min. After permeabilization, the samples were washed three times with PBS, blocked with 5% normal goat serum and 2% bovine serum albumin (BSA) in PBS for 60 min. The samples were then incubated overnight at 4 °C with a primary antibody against HO-1 (1:200 dilution; Cat. No. 10701-1-AP, Proteintech, Manchester, UK) diluted in antibody dilution buffer (1% BSA in PBS). Samples were then incubated with an Alexa Fluor 488-conjugated secondary antibody (Thermo Fisher Scientific, Waltham, MA, USA). Fluorescence imaging was performed using a Leica Lightning super-resolution microscope operated with Leica Application Suite X (LAS X; Leica, Mannheim, Germany).

### 2.12. Statistical Analysis

Data are presented as mean ± SEM from three independent donors (*n* = 3). Statistical analysis was carried out by *t*-test using GraphPad Prism 5.0 software (GraphPad Software, Boston, MA, USA). A value of *p* < 0.05 was considered statistically significant (* *p* < 0.05, ** *p* < 0.01, *** *p* < 0.001).

## 3. Results

### 3.1. Heme Promotes Carbon Monoxide Formation During Oxidative Modification of LDL

**Figure 1:**
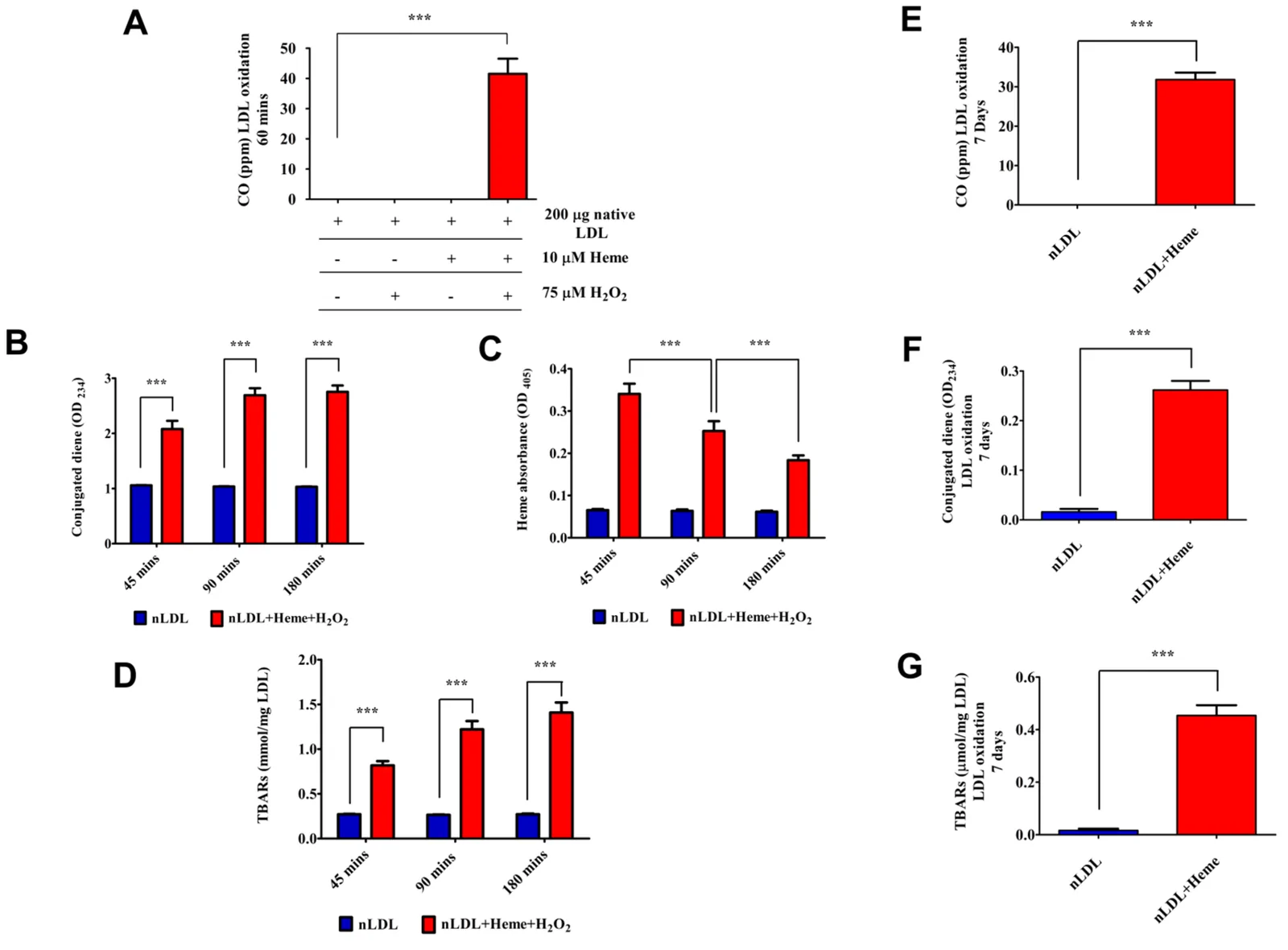
Heme promotes CO formation during oxidative modification of LDL. LDL (200 μg) isolated from three independent donors was incubated alone or with heme (10 μM), H_2_O_2_ (75 μM), or both heme and H_2_O_2_. Data are presented as mean ± SEM from three independent donors (*n* = 3). Statistical analysis was carried out by *t*-test. A value of *p* < 0.05 was considered statistically significant (*** *p* < 0.001). (**A**) CO production was measured with gas chromatography after 60 min of incubation (**B**) Conjugated diene formation was monitored at 234 nm for 180 min, and measurements obtained at 45, 90, and 180 min are presented. (**C**) Heme degradation was monitored by measuring the Soret absorbance at 405 nm (A_405_) for 180 min, and measurements obtained at 45, 90, and 180 min are presented. (**D**) Thiobarbituric acid-reactive substances (TBARs) were quantified after 45, 90, and 180 min. (**E**–**G**) LDL (200 μg) was incubated alone or with heme (10 μM) for 7 days. (**E**) CO production, (**F**) conjugated diene formation, and (**G**) TBARs levels were determined after 7 days of incubation.

To assess whether CO is generated during heme-induced LDL oxidation, LDL (200 μg) isolated from three donors was incubated alone or in the presence of heme (10 μM) and/or hydrogen peroxide (H_2_O_2_; 75 μM) for 60 min. CO release was undetectable in LDL incubated alone or with either heme or H_2_O_2_ individually (Figure 1A). In contrast, co-incubation with heme and H_2_O_2_ resulted in a significant increase in CO production (Figure 1A).

To evaluate lipid peroxidation, conjugated diene formation was monitored at 234 nm after 45, 90, and 180 min. Coincubation with heme and H_2_O_2_ significantly increased conjugated diene formation at all time points compared with LDL alone (Figure 1B). Because lipid peroxidation was markedly enhanced under these conditions, we next examined whether it was accompanied by heme degradation. Heme degradation was assessed by monitoring the Soret band at 405 nm. In the presence of LDL and H_2_O_2_, absorbance at 405 nm (A_405_) progressively declined over time, indicating the loss of spectroscopically intact heme (Figure 1C). To determine whether secondary lipid peroxidation products were also generated, thiobarbituric acid-reactive substances (TBARs) were quantified after 45, 90, and 180 min. Similar to conjugated diene formation, TBARs levels were significantly increased at all time points following co-incubation of LDL with heme and H_2_O_2_ (Figure 1D).

To determine whether CO generation also occurs during prolonged oxidation, LDL (200 μg) was incubated with heme (10 μM) for 7 days. CO release remained undetectable in LDL incubated alone, whereas incubation with heme resulted in a significant increase in CO production (Figure 1E). Consistent with these findings, prolonged exposure to heme also significantly increased conjugated diene (Figure 1F) and TBARs formation (Figure 1G), demonstrating sustained lipid peroxidation under these conditions.

### 3.2. CO Is Not Generated During CuSO_4_-Mediated Oxidation of LDL

**Figure 2:**
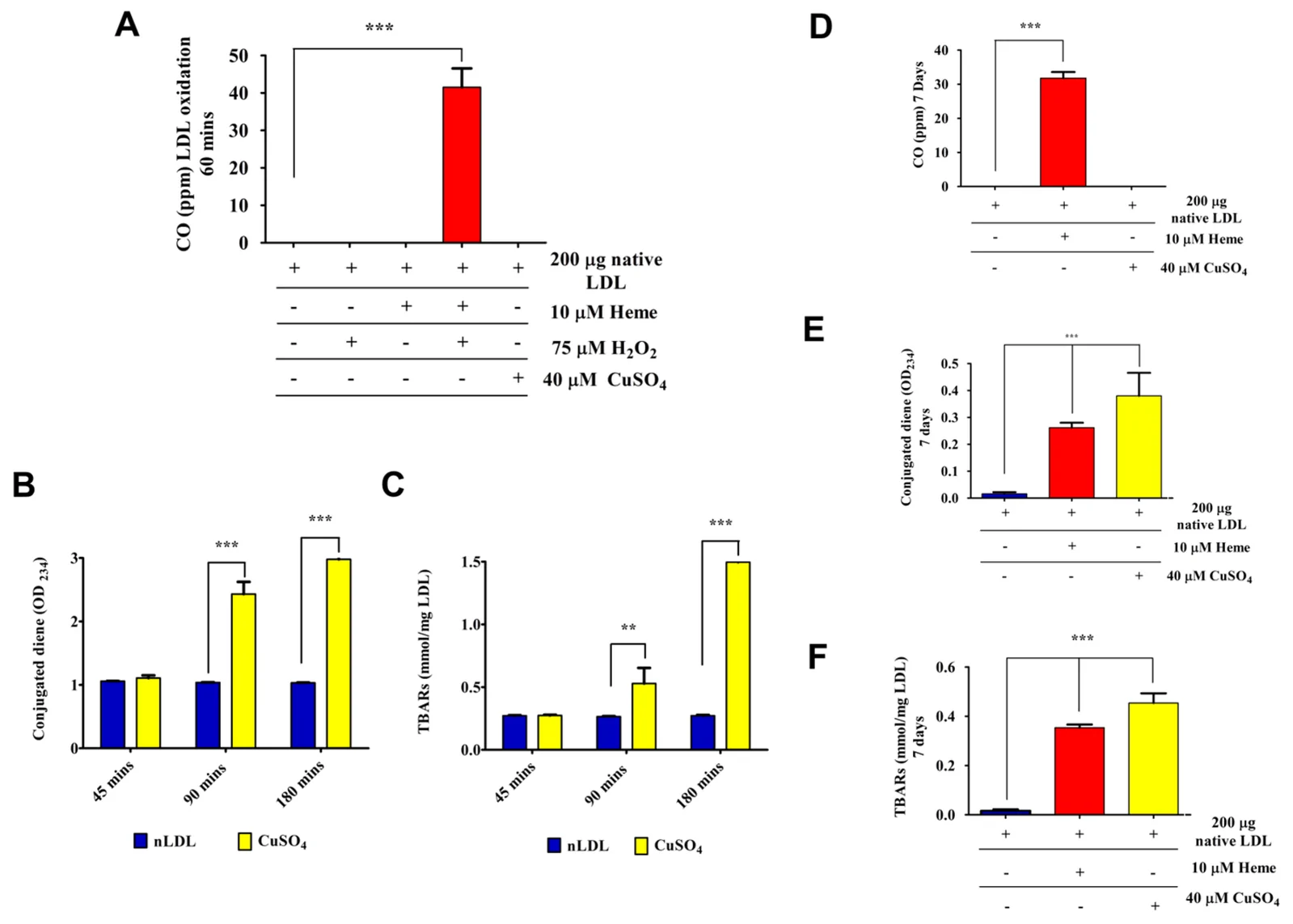
CuSO_4_-mediated oxidation of LDL does not induce CO formation. LDL (200 μg) was incubated alone or with CuSO_4_ (40 μM) under the indicated experimental conditions. Data are presented as mean ± SEM from three independent donors (*n* = 3). Statistical analysis was carried out by *t*-test. A value of *p* < 0.05 was considered statistically significant (** *p* < 0.01, *** *p* < 0.001). (**A**) CO production was measured after 60 min of incubation. (**B**) Conjugated diene formation, monitored at 234 nm, was determined after 45, 90 and 180 min of incubation. (**C**) Thiobarbituric acid-reactive substances (TBARs) were measured after 45, 90 and 180 min of incubation (**D**–**F**) LDL (200 μg) was incubated alone or with CuSO_4_ (40 μM) for 7 days. (**D**) CO production, (**E**) conjugated diene formation, and (**F**) TBARs levels were determined after 7 days incubation.

CuSO_4_ is a well-established in vitro catalyst of LDL oxidation. Therefore, we investigated whether CuSO_4_-mediated LDL oxidation is accompanied by CO formation. LDL (200 μg) was incubated alone or with CuSO_4_ (40 μM) for 60 min. CO release remained undetectable following CuSO_4_ treatment (Figure 2A). In contrast, CuSO_4_ significantly increased conjugated diene formation (Figure 2B) and TBARs levels (Figure 2C) after 90 min of incubation, confirming effective LDL oxidation.

To determine whether prolonged CuSO_4_-mediated oxidation induces CO formation, LDL (200 μg) was incubated with CuSO_4_ (40 μM) for 7 days. Similar to the short-term incubation, no CO release was detected after prolonged exposure to CuSO_4_ (Figure 2D). Nevertheless, CuSO_4_ significantly increased the formation of conjugated dienes (Figure 2E) and TBARs (Figure 2F) after 7 days of incubation, with levels exceeding those induced by heme, further confirming extensive oxidation of LDL in the absence of detectable CO production.

### 3.3. HO-1 Inhibition Reduces CO Formation and Delays Heme Degradation and Lipid Peroxidation in Heme-Oxidized LDL

**Figure 3:**
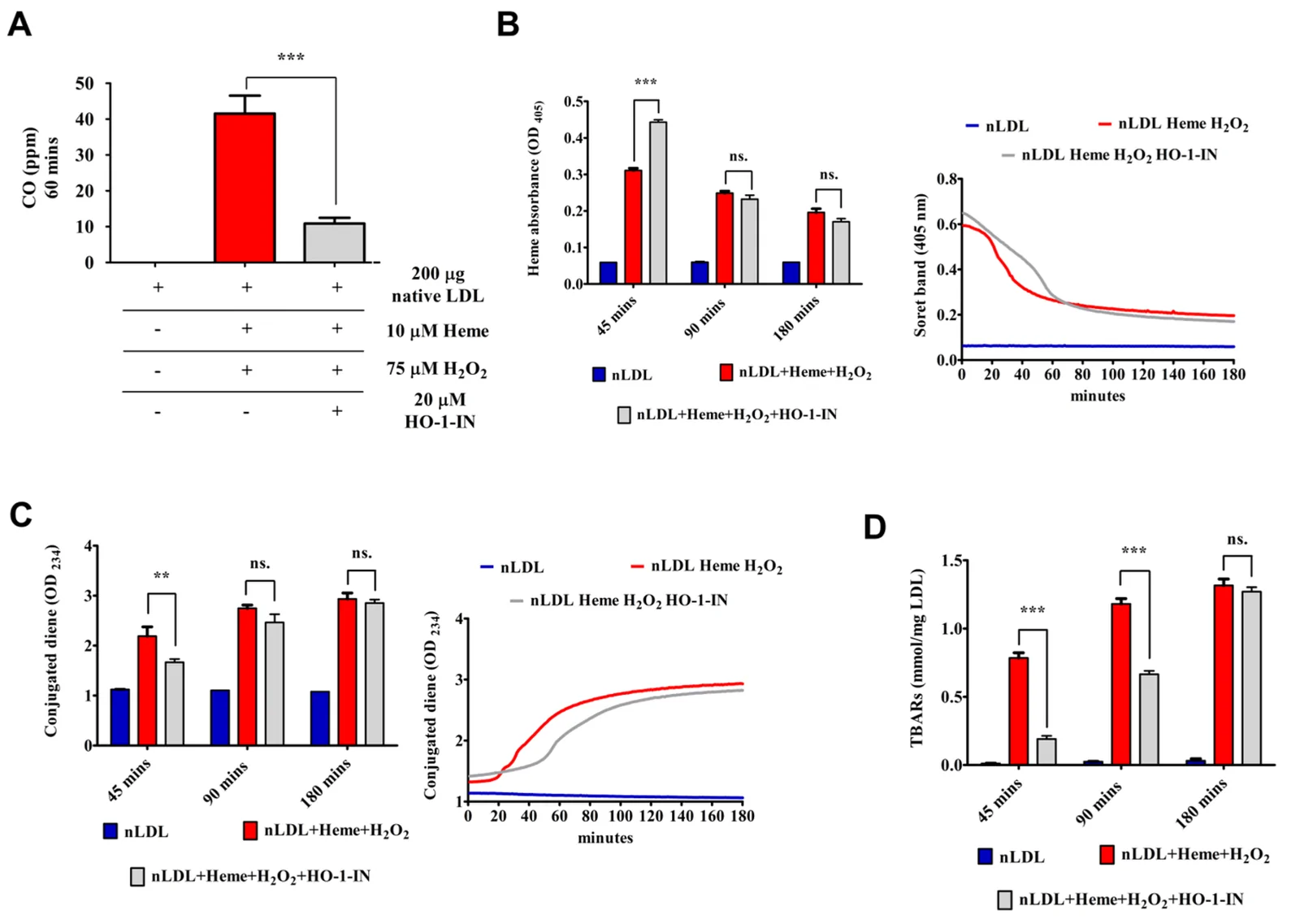
HO-1 Inhibition Reduces CO Formation and Delays Heme Degradation and Lipid Peroxidation in Heme-Oxidized LDL. LDL (200 μg) isolated from three independent donors was pre-incubated with HO-1-IN (20 μM), then further incubated with heme (10 μM) and H_2_O_2_ (75 μM). Data are presented as mean ± SEM from three independent donors (*n* = 3). Statistical analysis was carried out by *t*-test. A value of *p* < 0.05 was considered statistically significant (ns: non-significant, ** *p* < 0.01, *** *p* < 0.001). (**A**) CO production was measured after 60 min of incubation. (**B**) Heme degradation was assessed by monitoring the absorbance of the Soret band at 405 nm (A_405_) after 45, 90 and 180 min. (**C**) Conjugated diene formation, monitored at 234 nm, was determined after 45, 90 and 180 min of incubation. (**D**) Thiobarbituric acid-reactive substances (TBARs) were measured after 45, 90 and 180 min of incubation.

Next, we analyzed whether the HO-1 inhibitor, HO-1-IN inhibits CO generation. We showed HO-1-IN (20 μM) significantly decreased CO formation after 60 min generated by nLDL/heme/H_2_O_2_ (~40 ppm), but some residual (~10 ppm) CO production is still detectable (Figure 3A).

Next, we evaluated heme degradation in the presence of HO-1-IN over 45, 90, and 180 min. The LDL control remained stable throughout the experiment, indicating no appreciable time-dependent change (Figure 3B). In contrast, the Soret band intensity in the LDL/heme/H_2_O_2_ group gradually decreased over time, consistent with progressive heme degradation. At 45 min, the Soret band was significantly higher in the HO-1-IN-treated group, suggesting that HO-1-IN inhibited heme degradation (Figure 3B). However, by 90 and 180 min, the Soret band intensity in the HO-1-IN group was comparable to or slightly lower than that observed in the LDL/heme/H_2_O_2_ group (Figure 3B).

A similar trend was observed for conjugated diene formation. In the LDL/heme/H_2_O_2_ group, conjugated diene formation increased progressively over time, reaching its highest level at 180 min (Figure 3C). In contrast, the nLDL control remained stable throughout the experiment, indicating no appreciable time-dependent change (Figure 3C). At 45 min, conjugated diene formation was significantly reduced in the HO-1-IN-treated group compared with the nLDL/heme/H_2_O_2_ group (Figure 3C). However, by 90 and 180 min, conjugated diene formation in the HO-1-IN-treated group was comparable to or slightly lower than that in the LDL/heme/H_2_O_2_ group (Figure 3C).

TBARs formation exhibited a different kinetic profile. TBARs levels were significantly lower in the HO-1-IN-treated group than in the LDL/heme/H_2_O_2_ group at both 45 and 90 min. However, by 180 min, TBARs levels in the HO-1-IN-treated group were comparable to those in the LDL/heme/H_2_O_2_ group (Figure 3D).

### 3.4. Iron Chelation Decreases CO Formation and Delays Heme Degradation and Lipid Peroxidation in Heme-Oxidized LDL

**Figure 4:**
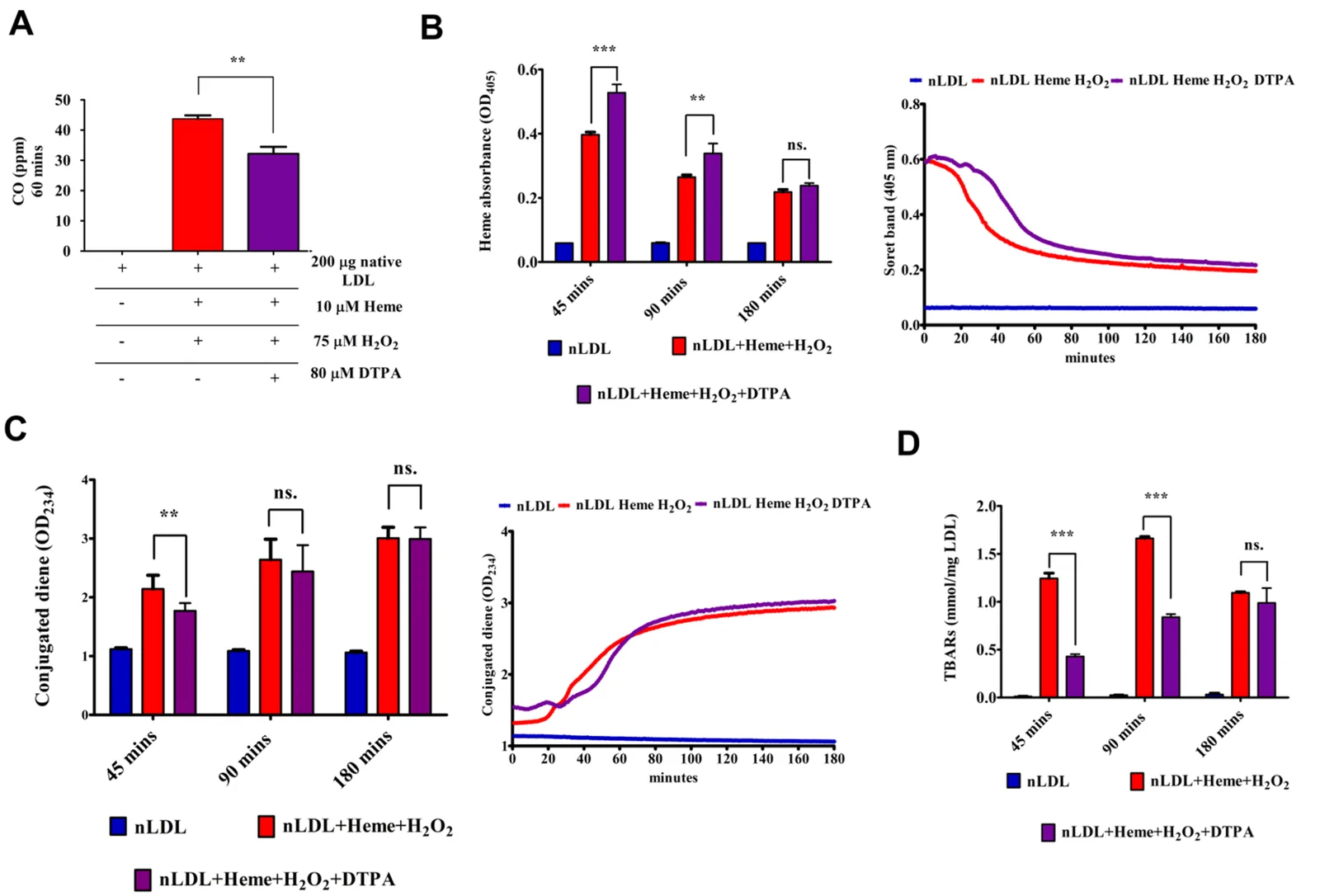
Iron Chelation Decreases CO Formation and Delays Heme Degradation and Lipid Peroxidation in Heme-Oxidized LDL. LDL (200 μg) isolated from three independent donors was pre-incubated with DTPA (80 μM) for 60 min, then further incubated with heme (10 μM) and H_2_O_2_ (75 μM). Data are presented as mean ± SEM from three independent donors (*n* = 3). Statistical analysis was carried out by *t*-test. A value of *p* < 0.05 was considered statistically significant (ns: non-significant, ** *p* < 0.01, *** *p* < 0.001). ns: non-significant (**A**) CO production was measured after 60 min of incubation. (**B**) Heme degradation was assessed by monitoring the absorbance of the Soret band at 405 nm (A_405_) after 45, 90 and 180 min. (**C**) Conjugated diene formation, monitored at 234 nm, was determined after 45, 90 and 180 min of incubation. (**D**) Thiobarbituric acid-reactive substances (TBARs) were measured after 45, 90 and 180 min of incubation.

Next, we analyzed whether the iron cheator, DTPA inhibits CO generation. We showed that DTPA (80 μM) significantly decreased CO formation after 60 min generated by nLDL/heme/H_2_O_2_ (~40 ppm), but some residual (~30 ppm) CO production was still detectable (Figure 4A).

Next, we examined the effect of iron chelation on heme degradation over 45, 90, and 180 min. The nLDL control remained stable throughout the experimental period, with no appreciable time-dependent change in Soret absorbance (Figure 4B). In contrast, Soret absorbance progressively decreased in the nLDL/heme/H_2_O_2_ group (Figure 4B). DTPA significantly attenuated the decrease in Soret absorbance at 45 and 90 min compared with the nLDL/heme/H_2_O_2_ group, indicating a delay in heme degradation (Figure 4B). By 180 min, Soret absorbance in the DTPA-treated group was no longer significantly different from that of the nLDL/heme/H_2_O_2_ group (Figure 4B).

Conjugated diene levels progressively increased in the nLDL/heme/H_2_O_2_ group, whereas the nLDL control remained stable throughout the experiment (Figure 4C). DTPA significantly reduced conjugated diene formation at 45 min. However, at 90 and 180 min, conjugated diene levels were not significantly different from those observed in the nLDL/heme/H_2_O_2_ group (Figure 4C), indicating that iron chelation delayed rather than prevented lipid peroxidation.

DTPA significantly reduced TBARS levels at 45 and 90 min compared with the nLDL/heme/H_2_O_2_ group, whereas this difference was no longer significant at 180 min (Figure 4D). Collectively, these findings indicate that iron chelation attenuates the early phase of heme-associated LDL oxidation but does not prevent the eventual development of lipid oxidation.

### 3.5. BHT Prevents CO Formation and Inhibits Heme Degradation and Lipid Peroxidation in Heme-Oxidized LDL

**Figure 5:**
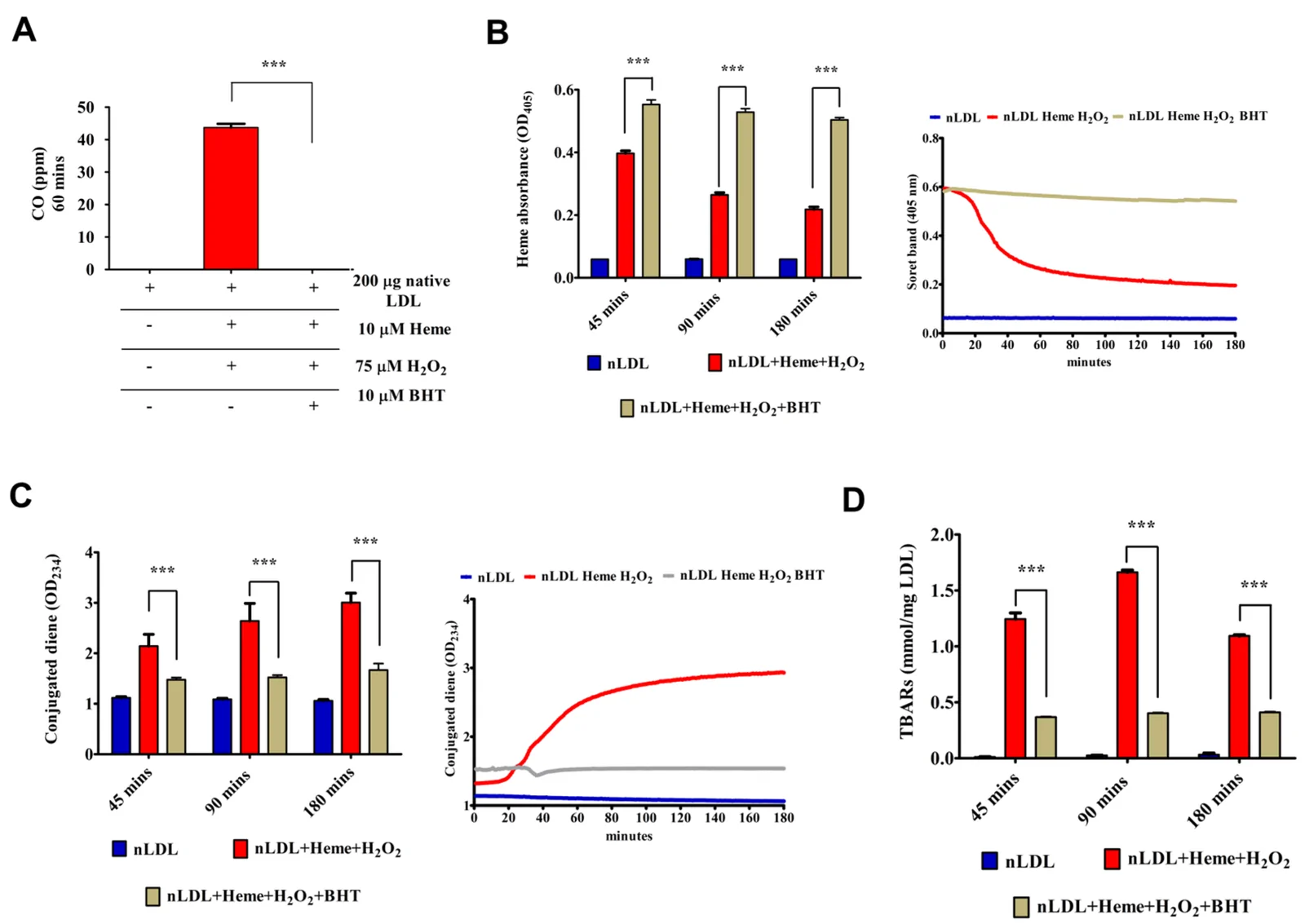
BHT Decreases CO Formation and Inhibits Heme Degradation and Lipid Peroxidation in Heme-Oxidized LDL. LDL (200 μg) isolated from three independent donors was pre-incubated with BHT (10 μM) for 60 min, then further incubated with heme (10 μM) and H_2_O_2_ (75 μM). Data are presented as mean ± SEM from three independent donors (*n* = 3). Statistical analysis was carried out by *t*-test. A value of *p* < 0.05 was considered statistically significant (*** *p* < 0.001). (**A**) CO production was measured after 60 min of incubation. (**B**) Heme degradation was assessed by monitoring the absorbance of the Soret band at 405 nm (A_405_) after 45, 90 and 180 min. (**C**) Conjugated diene formation, monitored at 234 nm, was determined after 45, 90 and 180 min of incubation. (**D**) Thiobarbituric acid-reactive substances (TBARs) were measured after 45, 90 and 180 min of incubation.

Because DTPA delayed, but did not prevent, lipid peroxidation, we next examined whether directly inhibiting lipid radical propagation with BHT inhibits heme/H_2_O_2_-driven LDL oxidation. We showed that BHT (10 μM) prevented CO formation after 60 min generated by nLDL/heme/H_2_O_2_ (Figure 5A).

In the LDL/heme/H_2_O_2_ group, heme absorbance progressively decreased over time, whereas the addition of BHT markedly preserved the Soret band intensity throughout the 180-min incubation period (Figure 5B). At 45, 90, and 180 min, heme absorbance was significantly higher in the BHT-treated group than in the LDL/heme/H_2_O_2_ group, indicating that BHT inhibited heme degradation (Figure 5B). In parallel, conjugated diene and TBARs formation increased progressively in the LDL/heme/H_2_O_2_ group, while BHT markedly attenuated this increase at all examined time points (Figure 5C,D). These findings demonstrate that inhibition of lipid peroxidation, the oxidative modification of LDL, is associated with preservation of heme, prevention a heme-degradation either by HO-1 or by direct interaction of heme with peroxides.

### 3.6. Identification of HO-1 and Heme-Binding Proteins in LDL Preparations

**Figure 6:**
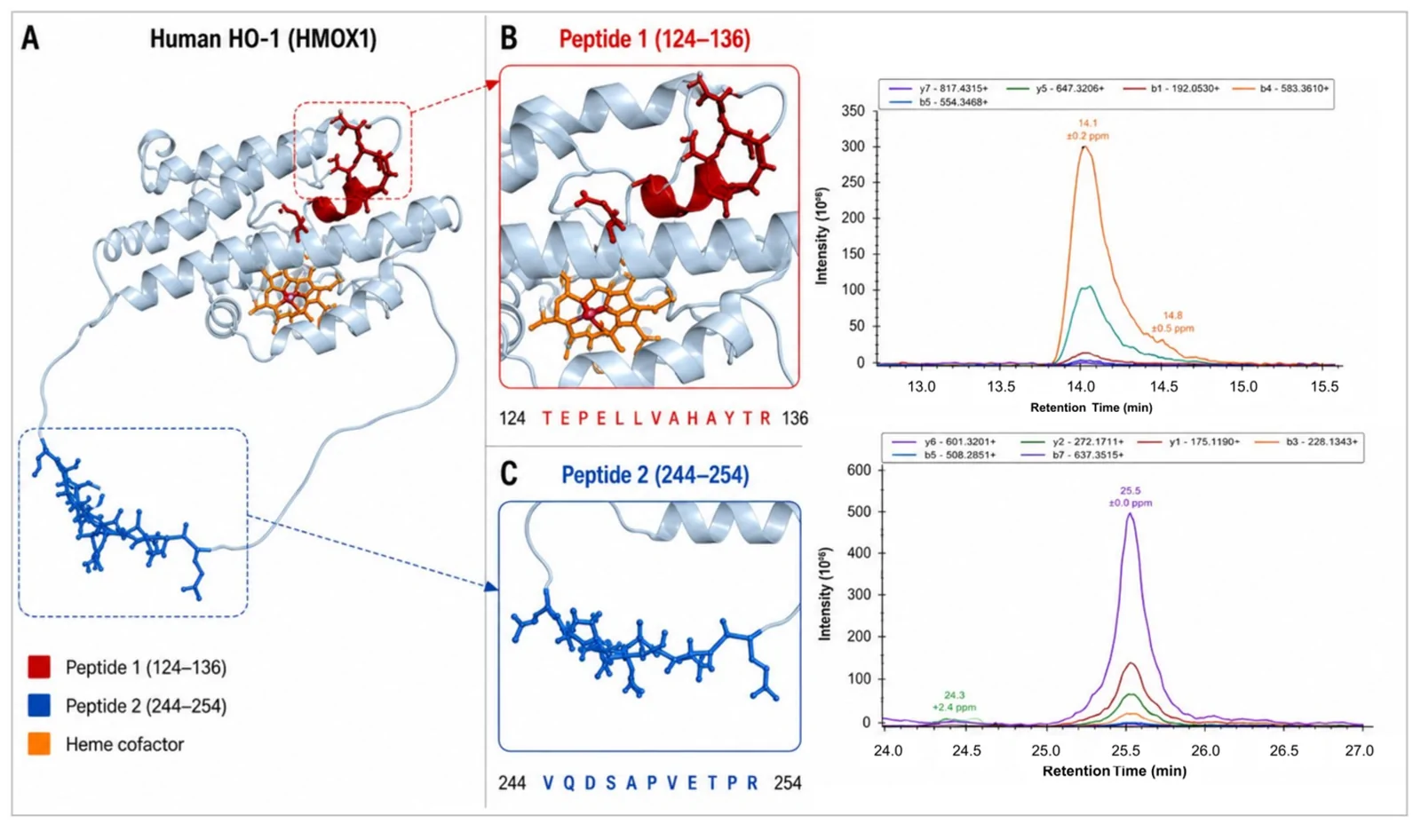
PRM-based targeted proteomics confirms the presence of HO-1 on LDL particles. (**A**) PRM-based targeted proteomics approach was used to verify the presence of HO-1 on LDL particles. Two proteotypic peptides were targeted: TEPELLVAHAYTR, located near the heme-binding pocket (**B**), and (**C**) VQDSAPVETPR, located within the C-terminal flexible loop.

Since the activity of HO-1 was proven by the functional assays, a PRM-based targeted proteomics experiment was carried out to prove the presence of this protein on LDL preparations (Figure 6A–C).

Two proteotypic peptides were analyzed: TEPELLVAHAYTR is located near the heme-binding pocket (Figure 6B) and VQDSAPVETPR in the C-terminal flexible loop (Figure 6C). As a result of the PRM-based targeted proteomics analysis, both TEPELLVAHAYTR and VQDSAPVETPR peptides were identified in the LDL preparations (Figure 6A–C), proving that LDL particles carry HO-1. For the examination of other potential heme-binding proteins on LDL particles, DIA-based proteomics experiments were carried out. The DIA analysis identified 72 proteins in the LDL samples with at least two identified tryptic peptides (Table 1). Among the 74 identified proteins, haptoglobin, hemopexin and alpha-1-microglobulin (A1M) were identified as heme-binding proteins.

**Figure 7:**
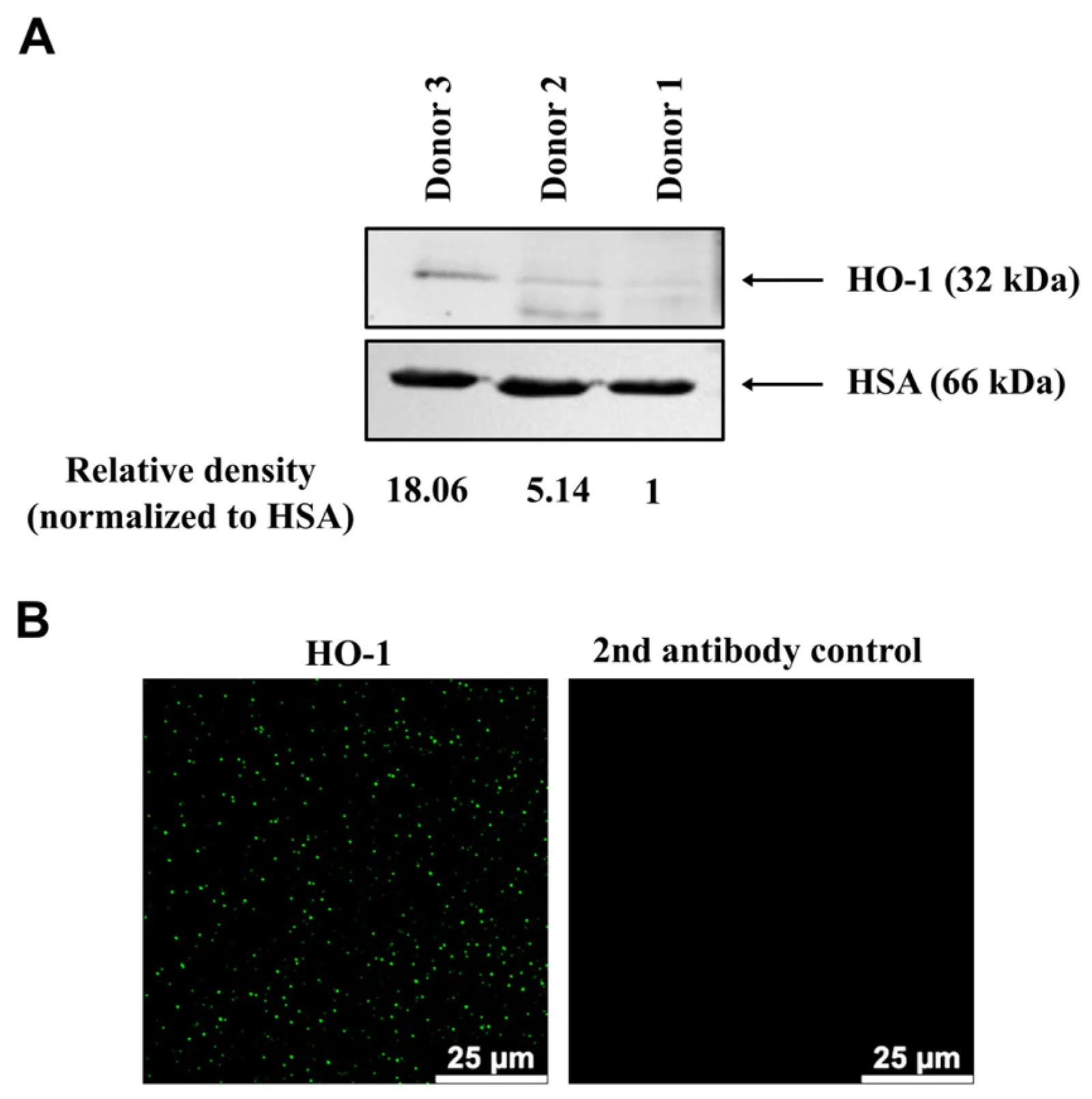
HO-1 is present in LDL preparations. Blood samples obtained from three independent donors (*n* = 3). (**A**) Representative immunoblot demonstrating the presence of HO-1 in nLDL preparations from three independent donors (*n* = 3). Original images can be found in Supplementary File S1. (**B**) Representative immunofluorescent analysis of HO-1 in LDL preparations.

To corroborate the presence of HO-1 in LDL preparation from the three donors, HO-1 protein was detected by immunoblotting in all donors, demonstrating immunoreactivity in each LDL preparations (Figure 7A). This finding was further supported by immunofluorescence staining, which also demonstrated HO-1 immunoreactivity in the LDL preparations (Figure 7B).

### 3.7. Hemopexin Inhibits Heme-Dependent CO Generation and LDL Oxidation

**Figure 8:**
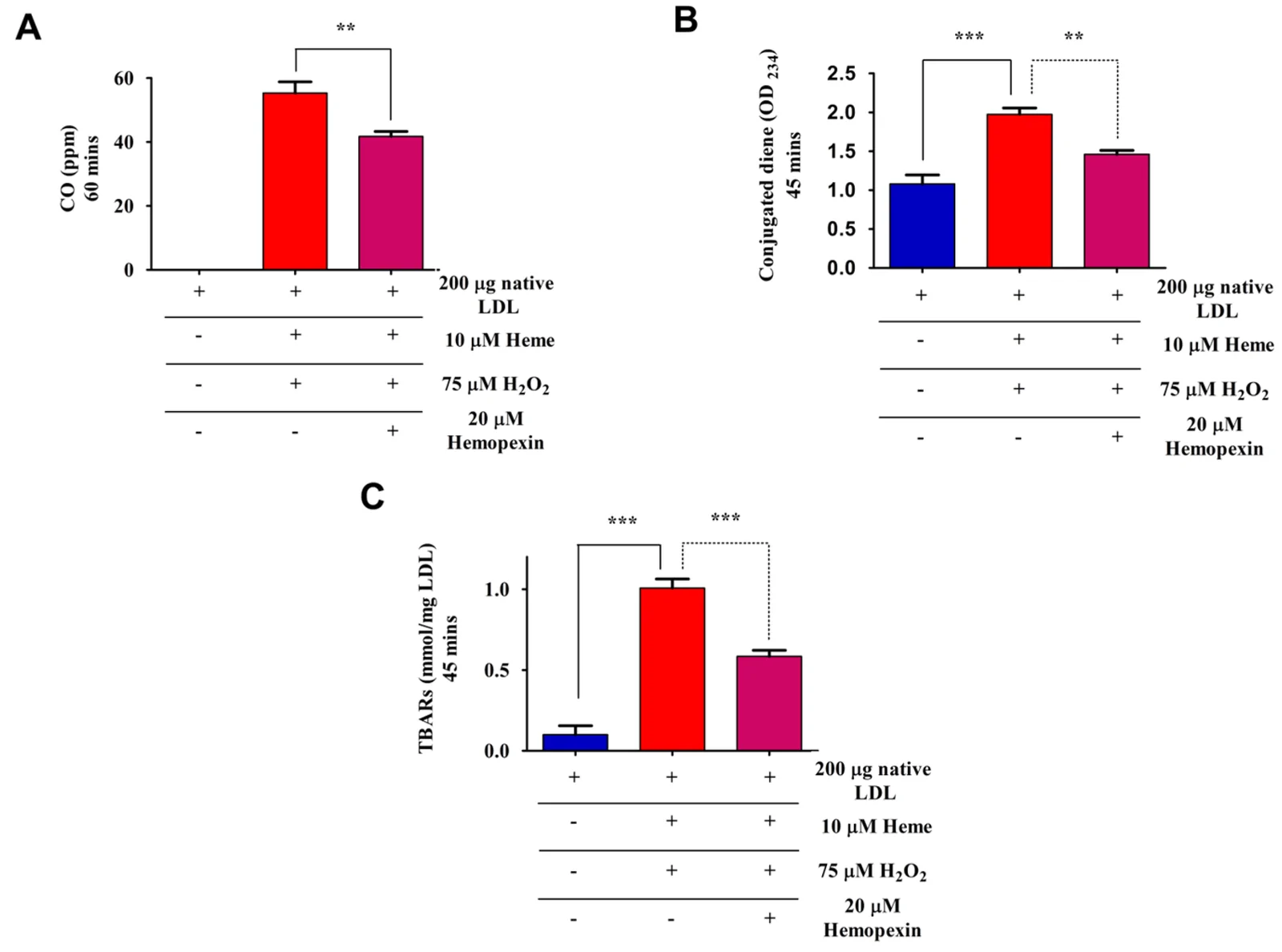
Hemopexin inhibits heme-dependent CO production and LDL oxidation. LDL (200 μg) was incubated with heme (10 μM) and H_2_O_2_ (75 μM) in the absence or presence of hemopexin (20 μM). Data are presented as mean ± SEM from three independent donors (*n* = 3). Statistical analysis was carried out by *t*-test. A value of *p* < 0.05 was considered statistically significant (** *p* < 0.01, *** *p* < 0.001). (**A**) CO production was quantified after 60 min using gas chromatography. (**B**) Conjugated diene formation and (**C**) TBARs levels were measured after 45 min.

Next, we investigated whether the heme-scavenging protein hemopexin inhibits CO formation. CO production induced by the co-incubation of LDL (200 μg) with heme (10 μM) and H_2_O_2_ (75 μM) was significantly inhibited in the presence of hemopexin (20 μM) (Figure 8A). Furthermore, hemopexin significantly reduced the formation of both conjugated dienes (Figure 8B) and TBARs (Figure 8C).

### 3.8. Heme Scavenger A1M Is Present in LDL Preparations and Inhibits LDL Oxidation

**Figure 9:**
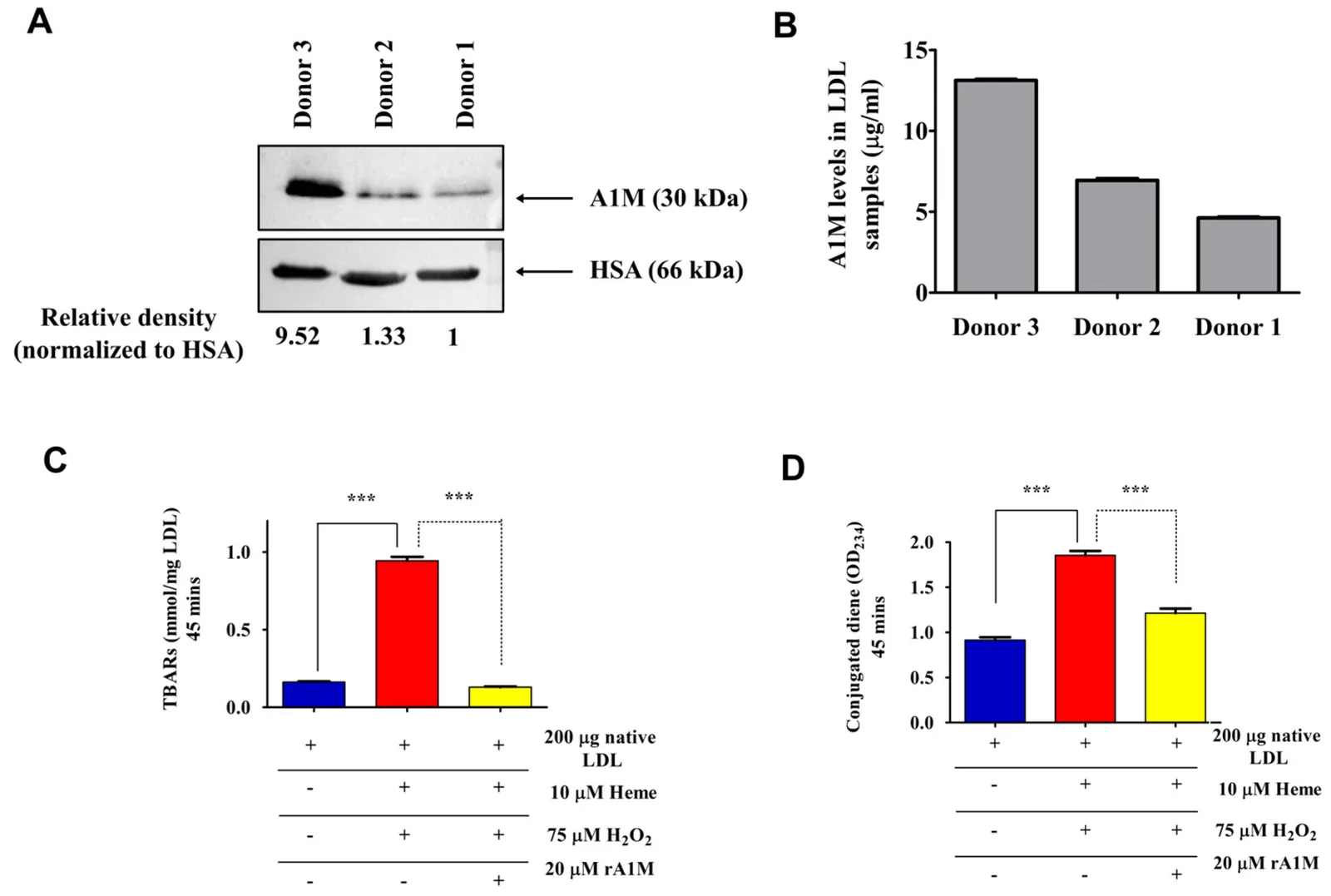
(**A**) Representative immunoblot demonstrating the presence of A1M in nLDL preparations from three independent donors. (*n* = 3) Original images can be found in Supplementary File S1. (**B**) ELISA measurement of A1M in nLDL preparations from three independent donors (*n* = 3). (**C**,**D**) LDL (200 μg) was incubated with heme (10 μM) and H_2_O_2_ (75 μM) in the absence or presence of A1M (20 μM). Data are presented as mean ± SEM from three independent donors (*n* = 3). Statistical analysis was carried out by *t*-test. A value of *p* < 0.05 was considered statistically significant (*** *p* < 0.001). (**C**) Conjugated diene formation and (**D**) TBARs levels were measured after 45 min.

To corroborate the presence of A1M in LDL preparation from the three donors, A1M protein was detected by immunoblotting in all donors, demonstrating immunoreactivity in each LDL preparations (Figure 9A). This finding was further supported by ELISA, which also demonstrated HO-1 immunoreactivity in the LDL preparations (Figure 9B).

To assess whether A1M inhibits heme-induced LDL oxidation, LDL (200 μg) isolated from three donors was incubated alone or in the presence of heme (10 μM) and H_2_O_2_ (75 μM) and A1M (20 μM) for 45 min. Coincubation with heme and H_2_O_2_ significantly increased conjugated diene formation that was attenuated by A1M (Figure 9C). To determine whether secondary lipid peroxidation products were also generated, thiobarbituric acid-reactive substances (TBARs) were quantified after 45 min. Similar to conjugated diene formation, TBARs levels were significantly increased at all time points following co-incubation of LDL with heme and H_2_O_2_ that was significantly decreased by A1M (Figure 9D).

## 4. Discussion

The principal finding of this study is the demonstration that native LDL contains detectable HO-1 and A1M, together with evidence that heme and H_2_O_2_ promote CO release during the oxidative modification of LDL. Although HO-1 is well established as a cytoprotective enzyme that degrades pro-oxidant heme, its association with circulating LDL has not been widely explored. Our findings therefore raise the possibility that LDL-associated heme-regulatory proteins contribute to the balance between oxidative damage and antioxidant defense within the vascular compartment.

Lipid oxidation initiated by reactive oxygen species (ROS) plays a significant role in atherosclerosis [7,24], and heme, as a hydrophobic iron-containing complex, acts as a catalyst in this process [25]. Our results demonstrate that heme and H_2_O_2_ synergistically promote LDL lipid oxidation, as indicated by increased conjugated diene formation. This effect was consistently observed in LDL isolated from three independent donors, and similar oxidation kinetics were obtained when the data were pooled, highlighting the reproducibility of the response. In contrast, neither heme nor H_2_O_2_ alone induced lipid oxidation, suggesting that both are required to initiate this process under the experimental conditions. This is in a good agreement with previous studies [7]. Collectively, our results demonstrate that heme in combination with H_2_O_2_ induces rapid and progressive oxidative modification of nLDL. This is evidenced by early conjugated diene formation and the subsequent accumulation of secondary lipid oxidation products (TBARs). In parallel, a time-dependent decrease in the Soret band indicates progressive heme degradation during the reaction, suggesting that heme is consumed during oxidative turnover. In contrast, neither heme nor H_2_O_2_ alone induced lipid oxidation or heme degradation under the conditions tested, highlighting the requirement for their combined presence.

Although CuSO_4_ ultimately produced a comparable extent of TBARs formation at later time points, its effects were delayed relative to the heme/H_2_O_2_ system, which induced earlier onset of lipid peroxidation. Together, these findings suggest that heme acts both as a catalyst and as a substrate in the presence of H_2_O_2_, promoting efficient initiation and propagation of LDL lipid peroxidation while undergoing concurrent structural degradation. This supports a self-amplifying oxidative process in which heme-driven reactions accelerate the formation of lipid oxidation products.

Others have found that the liver microsomal NADPH-cytochrome P450 reductase can destroy heme and heme-containing proteins under laboratory conditions. The destruction involves attack on ferrous heme using oxygen-derived reactive species. In addition H_2_O_2_ was generated during the reaction and appears to participate in oxidation. This reaction results in the formation of propentdyopent and CO as heme degradation product [26]. Others have found that CO can be produced during iron-dependent lipid peroxidation independently of the classical heme oxygenase pathway [27]. Brain tissue showed the highest production of both CO and lipid peroxidation products, followed by kidney, lung, spleen, and blood. In contrast, liver, heart, intestine, and testes produced little or no CO or lipid peroxidation products, likely because of higher endogenous antioxidant capacity [27]. CO generation was confined to membrane-containing subcellular fractions, supporting the idea that oxidative degradation of membrane lipids is the source of CO [27]. In phenobarbital-induced rabbit liver microsomes, CO production was dependent on both NADPH and Fe^2+^, occurring under conditions that promote microsomal lipid peroxidation [28]. Zn-protoporphyrin IX, a potent HO inhibitor, did not inhibit CO formation, demonstrating that the observed CO production was independent of the classical HO pathway [28]. Collectively, these studies demonstrate that CO can be generated through mechanisms independent of the classical HO pathway, particularly under conditions of iron-dependent lipid peroxidation and oxidative stress. Under conditions of increased oxidative stress, especially in tissues with limited antioxidant capacity, non-HO CO production may contribute significantly to endogenous CO levels, reflecting the extent of free radical-mediated lipid and heme oxidation rather than solely HO activity. In contrast, co-incubation of nLDL with heme and H_2_O_2_ resulted in a significant induction of CO release. CO release was undetectable in nLDL alone or following incubation with heme or H_2_O_2_ individually. Our results suggest that this CO production is parallel with heme degradation and oxidative stress in the LDL particles.

Given that HO-1 catalysis itself liberates ferrous iron as a stoichiometric byproduct alongside CO and biliverdin, and that free/released iron is a well-established catalyst of lipid peroxidation. Iron chelator DTPA and, to a lesser extent, desferrioxamine inhibits LDL oxidation when added during its initial stages indicating that iron availability influences the kinetics of LDL oxidation [29]. In line with this, the fungal iron chelator desferricoprogen diminishes oxidation of LDL and plaque lipids catalyzed by heme or hemoglobin [30]. Here we showed that DTPA delayed both heme degradation and conjugated diene formation, suggesting that redox-active iron contributes to the early progression of the oxidative process. The transient nature of this protection, however, indicates that iron chelation does not fully suppress oxidation. Rather, iron may facilitate the initiation of oxidative reactions, while subsequent lipid peroxidation and heme degradation can proceed through additional pathways that are less dependent on HO-1 and chelatable iron. This temporal pattern highlights a potential role for iron mobilization during heme degradation in amplifying LDL oxidation, while also indicating that released iron from porphyrin ring is not the sole mediator of the overall oxidative response, the heme-associated iron without liberation also contributes the oxidative modification of LDL.

The effects of BHT provide further evidence for an important interplay between lipid peroxidation and heme degradation during heme/H_2_O_2_-driven LDL oxidation. BHT strongly suppressed conjugated diene and TBARs formation, consistent with its ability to interrupt lipid peroxidation chain reactions. Notably, BHT also substantially preserved the heme Soret signal throughout the incubation period, suggesting that limiting lipid radical propagation can attenuate the subsequent loss of heme. This may suggest that the loss of the heme Soret signal is not attributable exclusively to HO-1-mediated enzymatic degradation, because BHT strongly prevented Soret loss despite the continued presence of HO-1. This suggests that oxidative modification/destruction of heme contributes substantially to the observed decrease in A_405_.

The temporal effects of HO-1 inhibition and iron chelation suggest that HO-1-associated iron release contributes to the early phase of heme-mediated LDL oxidation. LDL oxidation initiated by HO-1-dependent Fe^2+^ release from heme may subsequently become autocatalytic and less dependent on the initial HO-1-associated process. Indeed, once initiated, lipid peroxidation can propagate through radical-chain reactions in which lipid hydroperoxides generate additional reactive intermediates that promote further oxidation [31,32]. Consistent with this possibility, the restraining effect of HO-1 inhibition on lipid peroxidation diminished over time in our study, suggesting that mechanisms other than the initial HO-1-associated iron release may contribute to the propagation of oxidative damage. Furthermore, BHT strongly suppressed lipid peroxidation, preserved the heme Soret signal, and reduced CO production, supporting a close relationship between lipid peroxidation and heme degradation under our experimental conditions. Previous work has also demonstrated that oxidized LDL can induce HO-1 expression and activity in vascular smooth muscle cells [33], highlighting the interplay between LDL oxidation and cellular oxidative stress responses. Nevertheless, because HO-1 activity was not directly measured in the presence of BHT, we cannot exclude the possibility that BHT also affects heme degradation and CO production. Therefore, our findings are consistent with the possibility that autocatalytic lipid peroxidation contributes to the continued oxidative modification of LDL beyond the initial HO-1-associated phase, but they do not establish that subsequent LDL oxidation is entirely independent of HO-1 activity.

The DIA-based proteomics analysis revealed that LDL particles contain several proteins with known heme-binding properties, including haptoglobin, hemopexin, and A1M. These proteins are recognized for their roles in scavenging free hemoglobin and heme, thereby limiting their pro-oxidant and pro-inflammatory effects [8]. Their presence on LDL particles suggests that they may contribute to the interaction of LDL with heme under conditions of hemolysis or oxidative stress. Nevertheless, the potential contribution of these accessory heme-binding proteins to LDL-associated heme transport, sequestration, or oxidative modification warrants further investigation.

Previous studies have demonstrated that A1M inhibits myeloperoxidase-induced LDL oxidation, supporting a broader antioxidant role for A1M in the context of atherosclerosis [34]. In the present study, we showed that A1M is associated with LDL. Considering that A1M is a potent heme-scavenging protein with antioxidant and reductase properties [35], its ability to inhibit LDL oxidation in vitro is consistent with its known biological functions. Given these properties and its capacity to prevent oxidative modifications of LDL, A1M may represent an endogenous protective mechanism against the oxidative processes that contribute to the development and progression of atherosclerosis.

Although LDL is well characterized as a major carrier of cholesterol and lipids in the circulation, its protein composition remains incompletely understood. In this study, we identified HO-1 and A1M as previously unrecognized components of LDL, providing the first evidence for their presence within LDL particles. To the best of our knowledge, this study provides the first evidence for the presence of HO-1 and A1M in LDL.

Given the presence of the main heme-catabolizing enzyme, HO-1 in LDL particles, we investigated whether CO production involves HO-1 associated with LDL. In this work, we applied HO-1-IN as a selective HO-1 inhibitor, which belongs to the imidazole-type HO-1 inhibitors. HO-1 inhibition of imidazole-type inhibitors involves binding to the heme through the N atom of the imidazole, where imidazole nitrogen coordinates to ferric heme iron [36]. The present results demonstrate that HO-1-IN transiently attenuates heme degradation and the formation of heme-derived oxidation products. At 45 min, HO-1-IN preserved the Soret band intensity and significantly reduced conjugated diene and TBARs formation, indicating effective inhibition of the HO-1-mediated oxidative process during the early stages of incubation. However, this inhibitory effect diminished over time, as heme degradation and oxidation product formation in the HO-1-IN-treated group approached the levels observed in the nLDL/heme/H_2_O_2_ group by 90–180 min. Our results showed that HO-1-IN significantly delayed heme/H_2_O_2_-induced lipid peroxidation without altering the final extent of lipid oxidation, indicating that it primarily affects the kinetics of the oxidative process. The reduced efficacy at later time points may reflect incomplete or reversible enzyme inhibition, progressive loss of inhibitor activity under sustained oxidative conditions, or continued oxidative reactions that eventually overcome the inhibitory effect. Based on a previous finding on purified HO-1 [36], we hypothesize that the most plausible explanation for this is the reversible binding of imidazole-type inhibitors to human HO-1. Based on strong spectroscopic evidence, imidazole-containing HO-1 inhibitors, such as HO-1-IN used in this study, bind to and inactivate ferric HO-1 *via* noncovalent, reversible interactions [36]. Upon reduction to Fe^2+^, CO binds more strongly than the inhibitor’s imidazole nitrogen, which displaces the inhibitor from the heme iron [36]. Therefore, these inhibitors act through reversible, noncovalent coordination to the heme iron suggesting that HO-1-IN is possibly a ‘kinetic inhibitor’ under our experimental condition. Importantly, HO-1-IN significantly, although not completely decreased CO formation in LDL preparations in response to the combined treatment with heme and H_2_O_2_. However, we cannot exclude the possibility that heme catabolism associated with LDL occurs, at least in part, through HO-1-independent mechanisms.

Hemopexin binds heme in a 1:1 molar stoichiometry with one of the highest known affinities reported for a heme-binding protein (K_d_ ≈ 10^−13^ M) [37]. In plasma and other biological fluids, HPX-mediated sequestration of free heme is well established to protect against heme-catalyzed oxidative reactions, including LDL oxidation and endothelial cell injury [7,38]. The inhibitory effect of hemopexin on both CO production and lipid peroxidation supports the conclusion that free heme is required for CO generation during LDL oxidation. By tightly binding extracellular heme, hemopexin limits its pro-oxidant activity and thereby attenuates heme-catalyzed oxidative reactions [7]. The concomitant reduction in conjugated diene and TBARs formation further indicates that suppression of CO production is associated with decreased lipid peroxidation rather than a direct effect on CO itself. These findings reinforce the concept that heme availability is a key determinant of oxidative LDL modification and identify hemopexin as an effective inhibitor of this process.

## 5. Conclusions

This study provides new insights into the mechanisms regulating heme-mediated oxidative modification of LDL. Our findings demonstrate that inhibition of HO-1 significantly attenuates, but does not completely prevent, heme-induced lipid peroxidation, indicating that HO-1 contributes to, but is not solely responsible for, this process. The observation that the inhibitory effect diminishes over time is most consistent with reversible enzyme inhibition, although additional mechanisms cannot be excluded. Furthermore, we provide the first evidence that HO-1 and A1M are associated with LDL, expanding the current understanding of the protein composition of LDL particles and suggesting a previously unrecognized role in heme metabolism leading to CO release from LDL-associated heme. Nevertheless, the possibility that LDL-associated heme catabolism also occurs through HO-1-independent mechanisms warrants further investigation. HO-1-associated heme metabolism appears to contribute to the early phase of heme/H_2_O_2_-induced LDL oxidation, potentially in conjunction with iron-dependent redox chemistry. However, the persistent inhibition produced by BHT and the disappearance of the protective effects of HO-1 inhibition and iron chelation at later time points indicate that propagation of lipid oxidation is not dependent on continued HO-1 activity. Collectively, these findings advance our understanding of the interplay between heme metabolism and LDL oxidation and may have important implications for the pathogenesis of oxidative stress-related cardiovascular disease.

## Figures and Tables

**Table 1:** List of the identified proteins in LDL samples with their corresponding UniProt reference number. Heme-binding proteins are highlighted in bold.

Nr.	UniProt ID	Protein Name
1	P60709	Actin, cytoplasmic 1
2	P02763	Alpha-1-acid glycoprotein 1
3	P01011	Alpha-1-antichymotrypsin
4	P01009	Alpha-1-antitrypsin
5	P02765	Alpha-2-HS-glycoprotein
6	P01023	Alpha-2-macroglobulin
7	P02647	Apolipoprotein A-I
8	P02652	Apolipoprotein A-II
9	P06727	Apolipoprotein A-IV
10	P04114	Apolipoprotein B-100
11	P02654	Apolipoprotein C-I
12	P02655	Apolipoprotein C-II
13	P02656	Apolipoprotein C-III
14	P05090	Apolipoprotein D
15	P02649	Apolipoprotein E
16	Q13790	Apolipoprotein F
17	O14791	Apolipoprotein L1
18	O95445	Apolipoprotein M
19	P02749	Beta-2-glycoprotein 1
20	P49913	Cathelicidin antimicrobial peptide
21	P10909	Clusterin
22	P02746	Complement C1q subcomponent subunit B
23	P02747	Complement C1q subcomponent subunit C
24	P01024	Complement C3
25	P0C0L4	Complement C4-A
26	P07360	Complement component C8 gamma chain
27	P00746	Complement factor D
28	P36980	Complement factor H-related protein 2
29	P02671	Fibrinogen alpha chain
30	P02675	Fibrinogen beta chain
31	P02679	Fibrinogen gamma chain
32	P02751	Fibronectin
**34**	**P00738**	**Haptoglobin**
**35**	**P02790**	**Hemopexin**
36	P04196	Histidine-rich glycoprotein
37	Q86YZ3	Hornerin
38	P01876	Immunoglobulin heavy constant alpha 1
39	P01857	Immunoglobulin heavy constant gamma 1
40	P01859	Immunoglobulin heavy constant gamma 2
41	P01860	Immunoglobulin heavy constant gamma 3
42	P01591	Immunoglobulin J chain
43	P01834	Immunoglobulin kappa constant
44	P0DOX7	Immunoglobulin kappa light chain
45	P01597	Immunoglobulin kappa variable 1-39
46	P01602	Immunoglobulin kappa variable 1-5
47	P06310	Immunoglobulin kappa variable 2-30
48	P01619	Immunoglobulin kappa variable 3-20
49	A0A0A0MRZ8	Immunoglobulin kappa variable 3D-11
50	A0A087WSY6	Immunoglobulin kappa variable 3D-15
51	P06312	Immunoglobulin kappa variable 4-1
52	A0A0C4DH24	Immunoglobulin kappa variable 6-21
53	P0DOY3	Immunoglobulin lambda constant 3
54	A0M8Q6	Immunoglobulin lambda constant 7
55	P01700	Immunoglobulin lambda variable 1-47
56	P80748	Immunoglobulin lambda variable 3-21
57	P01717	Immunoglobulin lambda variable 3-25
58	A0A075B6H9	Immunoglobulin lambda variable 4-69
59	P0DOX8	Immunoglobulin lambda-1 light chain
60	P01042-2	Isoform LMW of Kininogen-1
61	P13647	Keratin, type II cytoskeletal 5
62	P02775	Platelet basic protein
63	Q9UHG3	Prenylcysteine oxidase 1
64	A0A075B6R9	Probable non-functional immunoglobulin kappa variable 2D-24
**65**	**P02760**	**Protein AMBP (A1M)**
66	P07988	Pulmonary surfactant-associated protein B
67	P0DJI8	Serum amyloid A-1 protein
68	P35542	Serum amyloid A-4 protein
69	P02743	Serum amyloid P-component
70	P27169	Serum paraoxonase/arylesterase 1
71	P02766	Transthyretin
73	P02774	Vitamin D-binding protein
74	P04004	Vitronectin

## Data Availability

The original contributions presented in this study are included in the article/Supplementary Materials. Further inquiries can be directed to the corresponding author.

## Supplementary Materials

The following supporting information can be downloaded at: https://www.mdpi.com/article/10.3390/biom16101500/s1. Supplementary File S1: The original images of the Western Blot and Raw data.

## Abbreviations

The following abbreviations are used in this manuscript:

A1M	Alpha-1-microglobulin
APOB-100	Apolipoprotein B-100
BHT	Butylated hydroxytoluene
CO	Carbon monoxide
CuSO_4_	Copper(II) sulfate
DMSO	Dimethyl sulfoxide
DTPA	Diethylenetriaminepentaacetic acid
Fe^2+^	Ferrous iron
H_2_O_2_	Hydrogen peroxide
HPX	Hemopexin
HO-1	Heme oxygenase-1
LDL	Low density lipoprotein
MS	Mass spectroscopy
NADPH	Nicotinamide adenine dinucleotide phosphate
nLDL	Native low density lipoprotein
rA1M	Recombinant alpha-1-microglobulin
ROS	Reactive oxygen species
TBARs	Thiobarbituric acid reactive substances
