# Supplementary material for: Carbon Monoxide Generation, Heme Turnover, and Lipid Peroxidation During Heme-Mediated LDL Oxidation: Association of HO-1, Hemopexin, and A1M with LDL
Source: Biomolecules. 2026 Oct 15;16(10):1500. doi: 10.3390/biom16101500 (PMC13702309; doi:10.3390/biom16101500)
Supplement: Supplementary file 1 [file biomolecules-16-01500-s001.zip › Supplementary File S1.pdf]

biomolecules

MDPI

Article

Carbon monoxide generation, heme turnover, and lipid peroxidation during heme-mediated LDL oxidation: association of HO-1, hemopexin, and A1M with LDL

Dávid Pethő [1,2], Gergő Kalló [3], Éva Csősz [3], István Bak [4], Magnus Gram [5], József Balla [1,2*], György Balla [2,6†] and Tamás Gáll [1,2†]

[1] Department of Internal Medicine, Division of Nephrology, Faculty of Medicine, University of Debrecen, H-4032, Debrecen, Hungary
[2] HUN-REN–UD Vascular Biology and Myocardium Pathophysiology Research Group, Hungarian Academy of Sciences, University of Debrecen, H-4032 Debrecen, Hungary
[3] Department of Biochemistry and Molecular Biology, Faculty of Medicine, University of Debrecen, H-4032, Debrecen, Hungary
[4] Department of Pharmacodynamics, Faculty of Pharmacy, University of Debrecen, Debrecen, Hungary
[5] Department of Clinical Sciences Lund, Pediatrics, Lund University, 221 84 Lund, Sweden
[6] Department of Pediatrics, Faculty of Medicine, University of Debrecen, H-4032, Debrecen, Hungary
† These authors share last authorship.
* Correspondence: balla@belklinika.com
Tel.: +36 52 255 600 / 55004
Department of Internal Medicine, Division of Nephrology, Faculty of Medicine, University of Debrecen, H-4032 Debrecen, Hungary

Original blots

Original blots of Figure 7A:

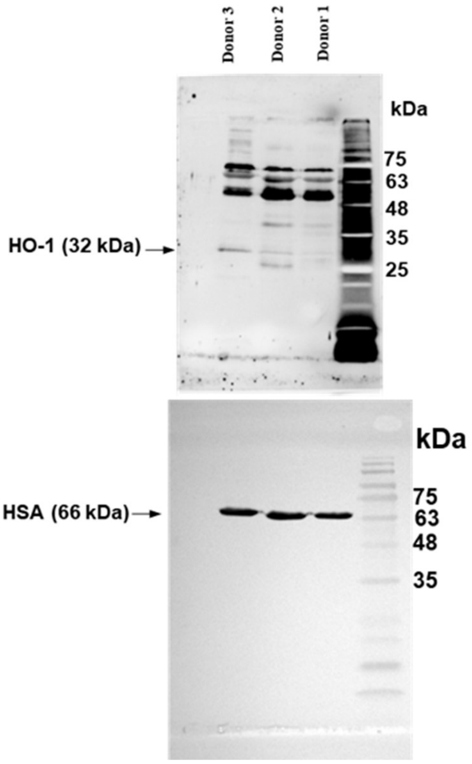

Original blots of Figure 9A:

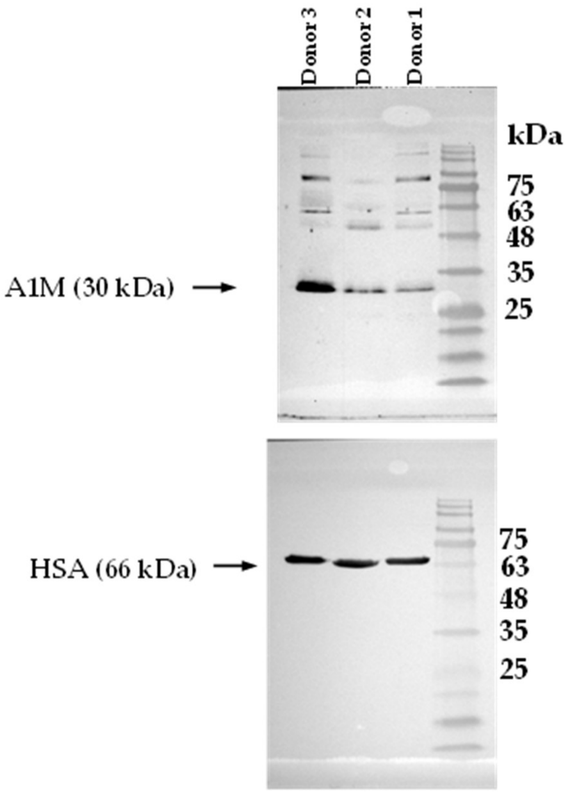

	nLDL	nLDL Heme	nLDL H2O2	nLDL Heme H2O2			
Figure 1.A.	0	0	0	45,8			
	0	0	0	30,8			
	0	0	0	55			
	0	0	0	47			
	0	0	0	29			
Figure 1.B.			nLDL			nLDL Heme H2O2	
	45 mins	1,05475	1,05575	1,06325	1,8345	2,349	2,05525
	90 mins	1,03025	1,036	1,04375	2,4995	2,93425	2,6435
	180 mins	1,02625	1,0315	1,03825	2,574	2,96625	2,724
Figure 1.C.			nLDL			nLDL Heme H2O2	
	45 mins	0,06275	0,07075	0,06325	0,3045	0,331	0,38625
	90 mins	0,0605	0,0705	0,05975	0,20775	0,26375	0,2865
	180 mins	0,058	0,067	0,05975	0,17625	0,206	0,169
Figure 1.D.			nLDL			nLDL Heme H2O2	
	45 mins	0,2719231	0,2783974	0,2654487	0,7833974	0,75837	0,9128846
	90 mins	0,2719231	0,2589743	0,2654487	1,197756	1,39199	1,074744
	180 mins	0,2848718	0,2654487	0,2654487	1,184808	1,52147	1,521474
Figure 1. E.	nLDL	nLDL Heme		Figure 1. F.	nLDL	nLDL Heme	
	0,04	35			0,01	0,27	
	0,01	37			0,01	0,28	
	0,01	28			0,03	0,2	
	0,02	29			0,03	0,31	
	0	30			0	0,25	
Figure 1. G.	nLDL	nLDL Heme					
	0,02	0,45					
	0,01	0,47					
	0,01	0,59					
	0,02	0,36					
	0,04	0,4					

Figure 2. A.	nLDL	nLDL Heme	nLDL H2O2	nLDL Heme H2O2	nLDL CuSO4		
	0	0	0	45,8	0		
	0	0	0	30,8	0		
	0	0	0	55	0		
	0	0	0	47	0		
	0	0	0	29	0		
Figure 2.B.			nLDL			nLDL CuSO4	
	45 mins	1,05475	1,05575	1,06325	1,081	1,0505	1,19025
	90 mins	1,03025	1,036	1,04375	2,172493	2,81058	2,3091
	180 mins	1,02625	1,0315	1,03825	2,49454	3,028	3,41625
Figure 2.C.			nLDL			nLDL CuSO4	
	45 mins	0,2719231	0,2783974	0,2654487	0,2654487	0,27192	0,2848718
	90 mins	0,2719231	0,2589743	0,2654487	0,2783974	0,65284	0,6541346
	180 mins	0,2848718	0,2654487	0,2654487	1,013237	1,47615	2,000577
Figure 2.D.	nLDL	nLDL+Heme	nLDL+CuSO4				
	0	35,1	0				
	0	37,2	0				
	0	28,8	0				
	0	29	0				
	0	30,6	0				
Figure 2. E.	nLDL	nLDL+Heme	nLDL+CuSO4				
	0,01	0,27	0,45				
	0,01	0,28	0,44				
	0,03	0,2	0,5				
	0,03	0,31	0,04				
	0	0,25	0,47				
Figure 2.F.	nLDL	nLDL+Heme	nLDL+CuSO4				
	0,01	0,4	0,45				
	0,01	0,35	0,47				
	0,03	0,32	0,59				
	0,03	0,347	0,36				
	0	0,35	0,4				

Figure 3. A.	nLDL	nLDL+Heme+ H2O2	nLDL+Heme+H2O2+HO1-IN						
	0	45,8	13,6						
	0	30,8	6,9						
	0	55	13,6						
	0	47	6,9						
	0	29	13,23						
Figure 3. B.	nLDL			nLDL+Heme+ H2O2			nLDL+Heme+H2O2+HO1-IN		
	0,059	0,05925	0,059	0,3045	0,3045	0,32325	0,45325	0,4303	0,445
	0,0603	0,05875	0,05975	0,2555	0,25325	0,2375	0,243	0,243	0,2108
	0,0595	0,0595	0,0595	0,206	0,206	0,17625	0,179	0,179	0,1533
Figure 3. C.	nLDL			nLDL+Heme+ H2O2			nLDL+Heme+H2O2+HO1-IN		
	1,123	1,124	1,123	1,8285	2,293	2,44875	1,605	1,602	1,797
	1,104	1,104	1,105	2,7325	2,86825	2,644	2,2025	2,7685	2,4198
	1,078	1,079	1,077	3,01275	3,092	2,69725	2,7425	2,9833	2,831
Figure 3. D.	nLDL			nLDL+Heme+ H2O2			nLDL+Heme+H2O2+HO1-IN		
	0,0067	0,01349359	0,01349359	0,7151603	0,7961218	0,84335	0,1619231	0,1754	0,2361
	0,027	0,02024039	0,02698718	1,113221	1,248157	1,18069	0,6341987	0,6477	0,7152
	0,0607	0,01349359	0,02024039	1,22117	1,356106	1,3696	1,288638	1,2077	1,3156
Figure 4.A.	nLDL	nLDL+Heme+ H2O2	nLDL+Heme+H2O2+DTPA						
	0	42,6	31,45115						
	0	46,6	40,92951						
	0	40,1	29,04665						
	0	43,7	31,00873						
	0	45,6	28,55						
Figure 4.B.	nLDL			nLDL+Heme+ H2O2			nLDL+Heme+H2O2+DTPA		
	0,059	0,05925	0,059	0,39925	0,38175	0,41	0,531	0,4823	0,5708
	0,0603	0,05875	0,05975	0,2665	0,276	0,25175	0,4	0,3065	0,3098
	0,0595	0,0595	0,0595	0,2185	0,2315	0,20475	0,25	0,2418	0,2225
Figure 4.C.	nLDL			nLDL+Heme+ H2O2			nLDL+Heme+H2O2+DTPA		
	1,123	1,0805	1,15375	1,7805	2,07575	2,5745	1,633	1,6428	2,0323
	1,0863	1,05475	1,12525	2,56425	2,07575	3,28025	2,4855	1,6428	3,1933
	1,062	1,0165	1,1	2,8215	2,8215	3,37525	2,7575	2,8388	3,3838
Figure 4.D.	nLDL			nLDL+Heme+ H2O2			nLDL+Heme+H2O2+DTPA		
	0,0067	0,01349359	0,01349359	1,352564	1,203526	1,17628	0,474359	0,4199	0,391
	0,027	0,02024039	0,02698718	1,686539	1,676923	1,62372	0,8982372	0,833	0,7899
	0,0607	0,01349359	0,02024039	1,100961	1,07532	1,10577	1,245193	1,0096	0,7115
Figure 5.A.	nLDL	nLDL+Heme+ H2O2	nLDL+Heme+H2O2+BHT						
	0	42,6	0						
	0	46,6	0						
	0	40,1	0						
	0	43,7	0						
	0	45,6	0						
Figure 5.B.	nLDL			nLDL+Heme+ H2O2			nLDL+Heme+H2O2+BHT		
	0,059	0,05925	0,059	0,39925	0,38175	0,41	0,524	0,5715	0,563
	0,0603	0,05875	0,05975	0,2665	0,276	0,25175	0,51075	0,5495	0,525
	0,0595	0,0595	0,0595	0,2185	0,2315	0,20475	0,51525	0,49	0,5065
Figure 5.C.	nLDL			nLDL+Heme+ H2O2			nLDL+Heme+H2O2+BHT		
	1,123	1,0805	1,15375	1,7805	2,07575	2,5745	1,42625	1,5583	1,4483
	1,0863	1,05475	1,12525	2,56425	2,07575	3,28025	1,436	1,568	1,568
	1,062	1,0165	1,1	2,8215	2,8215	3,37525	1,4305	1,712	1,8645
Figure 5.D.	nLDL			nLDL+Heme+ H2O2			nLDL+Heme+H2O2+BHT		
	0,0067	0,01349359	0,01349359	1,352564	1,203526	1,17628	0,3766026	0,3638	0,3622
	0,027	0,02024039	0,02698718	1,686539	1,676923	1,62372	0,4070513	0,3926	0,4071
	0,0607	0,01349359	0,02024039	1,100961	1,07532	1,10577	0,4054487	0,4054	0,4199

Figure 8.A.	nLDL	nLDL+Heme+ H2O2	nLDL+Heme+H2O2+HPX
	0	65,8	42,6
	0	59,8	36,6
	0	55	40,1
	0	47	43,7
	0	49	45,6
Figure 8.B.	nLDL	nLDL+Heme+ H2O2	nLDL+Heme+H2O2+HPX
	1	2,1	1,5
	1,3	1,9	1,5
	0,8	1,77	1,6
	0,9	2,22	1,4
	1,4	1,88	1,3
Figure 8.B.	nLDL	nLDL+Heme+ H2O2	nLDL+Heme+H2O2+HPX
	0	0,96	0,62
	0,1	0,986	0,7
	0,3	0,84	0,5
	0,1	1,08	0,5
	0	1,17	0,6
Figure 9.B.	Donor 3	Donor 2	Donor 1
	12,99	6,66285	4,7163
	13,241	6,864095	4,536
	12,69	7,066285	4,9063
	13,001	7,186409	4,336
Figure 9.C.	nLDL	nLDL+Heme+ H2O2	nLDL+Heme+H2O2+rA1M
	0,15	0,87	0,12
	0,165	0,957	0,132
	0,1733	1,00485	0,1386
	0,15	0,87	0,12
	0,165	0,957	0,132
	0,1733	1,00485	0,1386
Figure 9.D.	nLDL	nLDL+Heme+ H2O2	nLDL+Heme+H2O2+rA1M
	0,8	1,8	1,145
	0,88	1,688	1,0465
	0,924	1,84924	1,257325
	0,9	1,81	1,145
	0,94	1,988	1,3165
	1,04	1,9924	1,37325
